# A 2.5 V, 2.56 ppm/°C Curvature-Compensated Bandgap Reference for High-Precision Monitoring Applications

**DOI:** 10.3390/mi13030465

**Published:** 2022-03-18

**Authors:** Guangqian Zhu, Zhaoshu Fu, Tingting Liu, Qidong Zhang, Yintang Yang

**Affiliations:** School of Microelectronics, Xidian University, Xi’an 710071, China; zhugq@stu.xidian.edu.cn (G.Z.); combfu@163.com (Z.F.); tingtingliu1997@163.com (T.L.); ytyang@xidian.edu.cn (Y.Y.)

**Keywords:** bandgap reference, curvature compensation, low temperature coefficient, BCD process, battery monitoring

## Abstract

This work presents a high-precision high-order curvature-compensated bandgap voltage reference (BGR) for battery monitoring applications. The collector currents of bipolar junction transistor (BJT) pairs with different ratios and temperature characteristics can cause greater nonlinearities in ΔV_EB_. The proposed circuit additionally introduces high-order curvature compensation in the generation of ΔV_EB_, such that it presents high-order temperature effects complementary to V_EB_. Fabricated using a 0.18 µm BCD process, the proposed BGR generates a 2.5 V reference voltage with a minimum temperature coefficient of 2.65 ppm/°C in the range of −40 to 125 °C. The minimum line sensitivity is 0.023%/V when supply voltage varies from 4.5 to 5.5 V. The BGR circuit area is 382 × 270 μm^2^, and the BMIC area is 2.8 × 2.8 mm^2^.

## 1. Introduction

**Figure 1:**
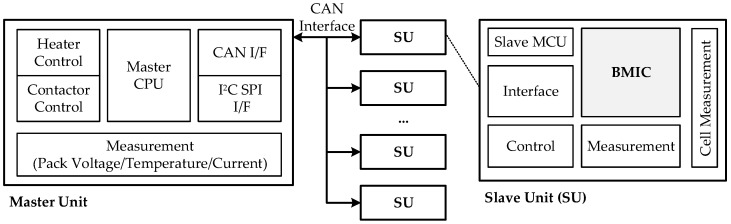
The structure of the proposed BMS.

The battery management system (BMS) guarantee the working performance and service life of the battery, and provides new energy management for various applications such as electric vehicles (EVs), energy storage system, and aerospace satellites. Battery monitoring (including voltage, current, temperature, State of Charge (SOC), State of Health (SOH)) is the most basic and core application of the BMS. In typical BMS, it is necessary to constantly evaluate various parameters pertaining to Li-ion battery packs. Monitoring precision is the fundamental guarantee for the reliability and performance of EVs. Figure 1 presents the structure of the proposed BMS.

**Figure 2:**
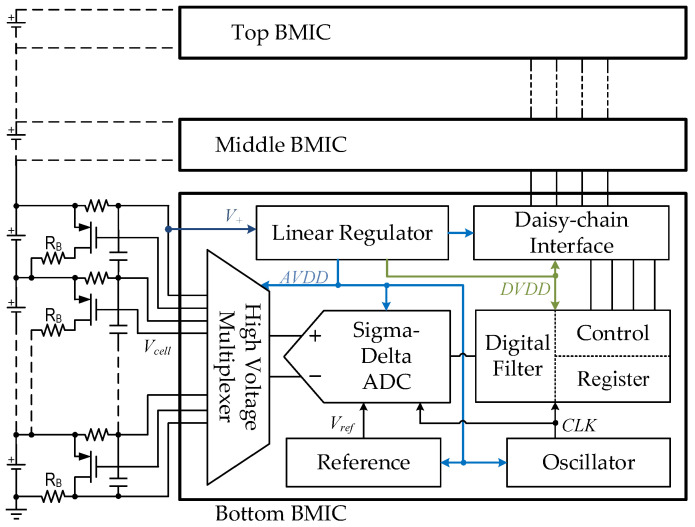
The structure of the proposed BMIC.

“One master, many slaves” architecture is used for our BMS. The master unit (MU) mainly measures the total voltage, total current, pressure and collision information of the battery pack, calculates the SOC and SOH values, and controls multiple slave units (SUs). The SUs mainly sense the voltage of each battery cell and the temperature of several points in the BMS box. Communication between MU and SUs is through a controller area network (CAN) interface. The battery monitoring integrated circuit (BMIC) is the most significant device used in the BMS slave unit. It connects directly to the battery pack and is designed to monitor multiple cell voltages and temperatures [1,2]. Figure 2 presents the structure of the proposed BMIC.

In order to achieve a high-integration and low-power BMS design as much as possible, a multiplexer structure is used in the BMIC to expand the number of measurable battery cells, and then a high-precision reference circuit and ADC are used to ensure the measurement accuracy. In this structure, the monitoring error mainly comes from the error on the multi-channel sensing channel, the ADC error and the reference error. The accuracy of the voltage reference is the most important criterion that determines the precision of battery monitoring, and it is also an indispensable part in other precision sensors or applications [3,4,5]. 

Bandgap voltage reference (BGR) is the mainstream temperature- and voltage- insensitive reference used in the field. In high-precision BGR applications, the use of only first-order linear compensation [6,7,8,9,10,11,12] is insufficient for achieving the required temperature coefficient (TC). Effective and simple high-order temperature compensation methods have thus become the norm for optimized circuit design. The curvature-compensation methods reported in the literature [13,14,15,16,17,18,19,20,21,22] further offset the temperature nonlinearity of the emitter–base voltage (V_EB_). The topologies in [13] combine opposing curvature characteristics produced by the two BGR cores to achieve the reference voltage. However, even with high-order temperature compensation, the output voltage inevitably drifts owing to factors such as process spread, device aging, and stress. Therefore, the on-chip calibration or trimming structure after production is important to ensure accuracy. The BGR circuits in [14,16] were designed specifically for battery management applications; specifically, the switched-capacitor bandgap reference in [14] and its high-order compensation are achieved by replacing the analog circuitry with a more sophisticated digital correction algorithm [15]. The internal temperature sensor and a lookup table will incur additional cost. The topologies in [16] have piecewise exponential curvature compensation such that good temperature characteristics can be obtained over a wide temperature range, however, the compensation structure is slightly complicated. A zero TC biased MOSFET compensation method is used in [17], but the untrimmed reference voltage is greatly affected by process spread. The circuit in [18] is an ultra-low-power BGR structure, but the TC of the reference voltage is extremely large.

This paper presents a V_EB_-based high-order curvature- compensated BGR with a low TC over a temperature range of −40 to 125 °C. The designs in [19] exploit different collector currents to enable logarithmic curvature compensation of ΔV_EB_. Based on the idea, a new ΔV_EB_ generation structure is also proposed. The remainder of this paper is organized as follows. Section 2 illustrates the principle of the proposed BGR, and Section 3 presents the experimental results; the conclusions are presented in Section 4.

## 2. Principles of the Proposed BGR

### 2.1. Basic BGR Topologies

**Figure 3:**
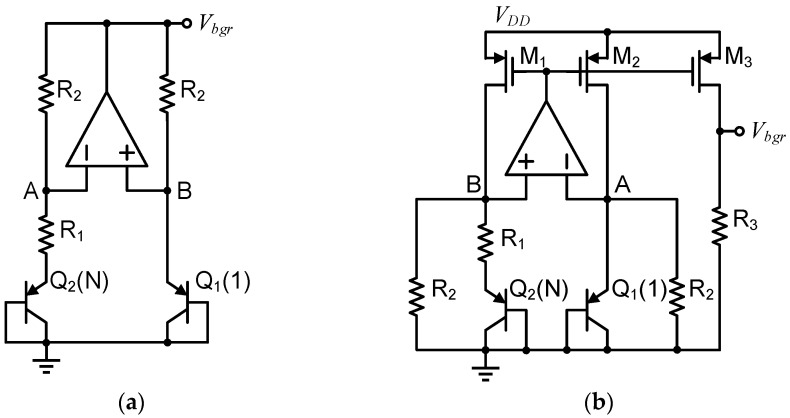
Schematic of the widely used BGR structures: (**a**) voltage-mode; (**b**) current-mode.

The V_EB_ of a bipolar junction transistor (BJTs) (or V_BE_ for an NPN transistor) is a complementary-to-absolute-temperature (CTAT) parameter with a TC of about −1.6 mV/°C, and the temperature dependence of V_EB_ [23] can be expressed as

(1) $$V_{EB}\left(T\right)=V_{G0}\left(T_{r}\right)-\underset{\mathrm{linear}}{\underbrace{\left[V_{G0}\left(T_{r}\right)-V_{EB0}\left(T_{r}\right)\right]T/T_{r}}}-\underset{\mathrm{nonlinear}}{\underbrace{V_{T}\left(\eta -\theta \right)\ln\left(T/T_{r}\right)}},$$

where V_G0_(T_r_) is the extrapolated bandgap voltage at a reference temperature T_r_, η is a temperature-insensitive parameter [24], and θ is the temperature dependence order of the collector current. Figure 3 shows two widely used BGR structures based on the first-order temperature compensation.

The bandgap voltage V_bgr_ of the voltage-mode BGR [6] in Figure 3a is given by

(2) $$V_{bgr}=V_{EB}+\frac{R_{2}}{R_{1}}\Delta V_{EB}=V_{EB}+\frac{R_{2}}{R_{1}}\cdot \frac{kT}{q}\ln\left(N\right),$$

and the V_bgr_ of the current-mode BGR [7] in Figure 3b is given as

(3) $$V_{bgr}=R_{3}\left(\frac{V_{EB}}{R_{2}}+\frac{\Delta V_{EB}}{R_{1}}\right)=\frac{R_{3}}{R_{2}}V_{EB}+\frac{R_{3}}{R_{1}}\cdot \frac{kT}{q}\ln\left(N\right),$$

where V_T_ = kT/q is the thermal voltage with a TC of about 85 μV/°C, k is the Boltzmann constant, q is the electron charge, and *N* is the emitter-area ratio of Q_2_ to Q_1_.

First-order compensation can only decrease the TC of V_ref_ to about 13 ppm/°C in the presence of nonlinearity [6,7,8,9,10,11,12]. In high-precision battery monitoring, it is necessary to detect voltage changes below 3 mV, and the TC of the reference voltage must be less than or equal to 6 ppm/°C [16]. Therefore, further reduction of the TC requires compensation of the higher-order terms related to Tln(T) in V_EB_.

### 2.2. Insertion of Nonlinear Compensation in ΔV_EB_

The current-mode or voltage-mode BGRs primarily use the proportional-to-absolute-temperature (PTAT) characteristic of ΔV_EB_, where ΔV_EB_ is expressed as

(4) $$\Delta V_{EB}=V_{EB1}-V_{EB2}=\frac{kT}{q}\ln\left(N\cdot \frac{I_{c1}}{I_{c2}}\right).$$

If the collector currents I_c1_ and I_c2_ of the PNP BJT pair (Q_1_ and Q_2_) have the same temperature characteristics, and ΔV_EB_ is a more easily controllable linear compensation term. However, if the TC of collector currents are different, then a nonlinear term is introduced into ΔV_EB_ through the logarithm function. 

**Figure 4:**
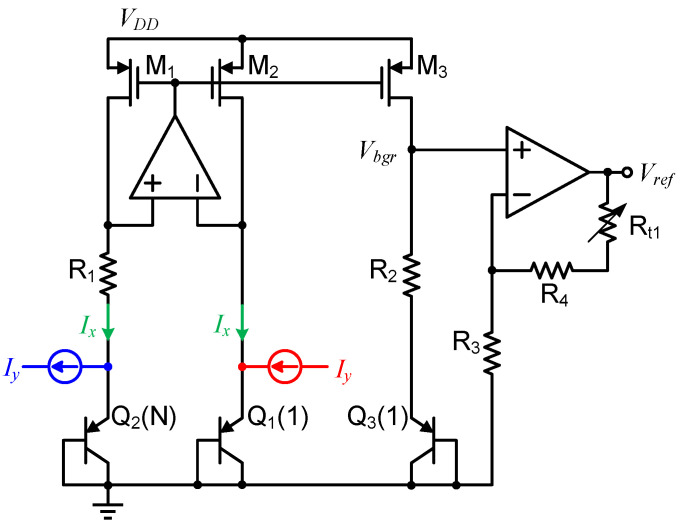
Principle of the proposed curvature compensation BGR.

Therefore, based on the conventional BGR structures in Figure 3, the principle of the curvature-compensated BGR in this work is shown in Figure 4. Based on the original bias current I_x_ of Q_1_ and Q_2_, the current I_y_ is introduced and drawn to form the difference in the collector currents of the BJT pair. I_x_ and I_y_ have different temperature characteristics, which lead to an increase in the nonlinearity of ΔV_EB_. Hence, ΔV_EB_ is rewritten as

(5) $$\Delta V_{EB}=V_{T}\cdot \ln\left(N\right)+V_{T}\cdot \ln\left(\frac{1+I_{y}/I_{x}}{1-I_{y}/I_{x}}\right).$$

Assuming that I_y_/I_x_ is a temperature-dependent function, i.e., x(T) = I_y_/I_x_. The natural logarithm has the Maclaurin series

(6) $$\ln\left(1+x\right)={\left(-1\right)}^{n+1}\sum_{n=1}^{\infty }\frac{x^{n}}{n}=x-\frac{x^{2}}{2}+\frac{x^{3}}{3}\cdots +{\left(-1\right)}^{n+1}\cdot \frac{x^{n}}{n},$$

which converges for |*x*| < 1. The logarithmic term in ΔV_EB_ can thus be calculated as

(7) $$\ln\left(\frac{1+x}{1-x}\right)=2\left(x+\frac{x^{3}}{3}+\frac{x^{5}}{5}+\frac{x^{7}}{7}+\cdots +\frac{x^{2n-1}}{2n-1}\right).$$

**Figure 5:**
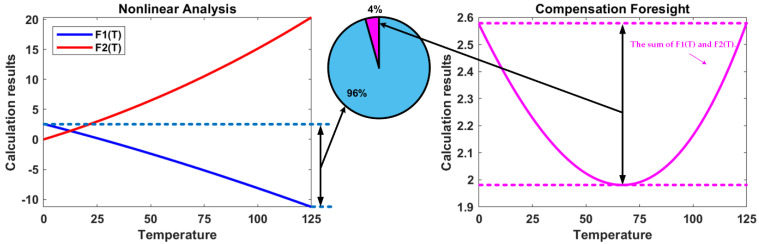
Ideal results calculated for the proposed curvature compensation.

From the Taylor expansion results, it is evident that the logarithmic function can compensate for the third-order term at least. It is worth noting that |x| < 1 is a necessary condition, so it must be guaranteed during circuit design. To simulate the compensation effects of (7) on the nonlinear term Tln(T) in V_EB_, construct the temperature-related functions F_1_(T) and F_2_(T) for the ideal calculations. F_1_(T) and F_2_(T) are expressed as

(8) $$\left\{ \begin{array}{l} F_{1}(T)=-r_{0}\cdot T\cdot \ln\left(\frac{T}{T_{r}}\right)\\ F_{2}(T)=r_{0}\cdot T\cdot \ln\left(\frac{1+h\cdot T}{1-h\cdot T}\right) \end{array}\right.,$$

where r_0_ is a constant, and h is a coefficient that ensures hT < 1. F_1_(T) and F_2_(T) were combined in different proportions to obtain the predicted compensation results shown in Figure 5.

It is observed that the deviation between the maximum and minimum values of F_1_(T) after compensating for F_2_(T) is only 4% of that before compensation, which better suppresses the nonlinear term in V_EB_ and realizes curvature compensation.

In Figure 4, after obtaining the ΔV_EB_ with high-order compensation effect, the ΔV_EB_ with the coefficient R_2_/R_1_ is obtained through R_1_, R_2_, M_1_ and M_3_, and it is added to the V_EB_ of Q_3_ to obtain the compensated bandgap voltage V_bgr_. In order to further obtain the required reference voltage, the final reference voltage V_ref_ is obtained through the negative feedback structure composed of R_3_, R_4_, R_t1_ and the amplifier. V_ref_ can be expressed as

(9) $$V_{ref}=\frac{R_{3}}{R_{3}+R_{4}+R_{t1}}\cdot V_{bgr}=\frac{R_{3}}{R_{3}+R_{4}+R_{t1}}\cdot \left(V_{EB}+a\cdot \frac{R_{2}}{R_{1}}\Delta V_{EB}\right),$$

where the parameters a is the size ratio of M_1_ and M_3_.

### 2.3. Implementation of the Proposed Circuit

**Figure 6:**
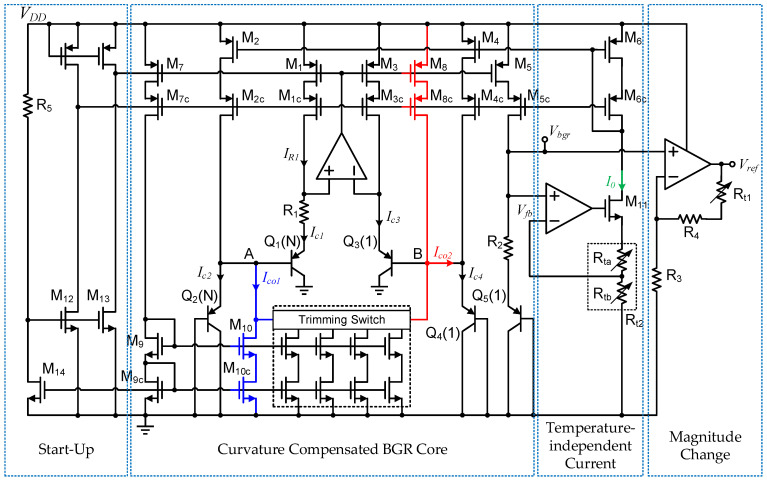
Schematic of the proposed curvature-compensated BGR circuit.

According to the ideal results obtained above, the main design goal is to introduce a nonlinear ΔV_EB_ in the BGR core circuit. Figure 6 presents the implementation of the proposed circuit, including a start-up circuit, a nonlinear ΔV_EB_-based curvature-compensated BGR core circuit, a temperature-independent current generating structure, and a final reference voltage output.

The R_5_ and NMOS M_12_, M_13_, M_14_ constitute a start-up circuit to drive the reference circuit out of the degenerate bias point when the supply voltage V_DD_ is turned on. When V_DD_ rises, M_12_ and M_13_ are turned on, and the gate voltage of the PMOS current mirrors is pulled down. After the whole circuit is started, M_14_ is turned on, and M_12_ and M_13_ are turned off.

The traditional scheme of ΔV_EB_ generation uses currents with the same temperature characteristics to drive a pair of BJTs. The main difference in the proposed nonlinear ΔV_EB_ generation unit is that two sets of currents with different temperature characteristics are used to drive two sets of BJTs (Q_1_ and Q_3_, Q_2_ and Q_4_). The ΔV_EB_ of the proposed BGR is then given as

(10) $$\Delta V_{EB}=V_{EB1}+V_{EB2}-V_{EB3}-V_{EB4}=2V_{T}\cdot \ln\left(N\right)+V_{T}\cdot \ln\left(\frac{I_{c3}}{I_{c1}}\right)+V_{T}\cdot \ln\left(\frac{I_{c4}}{I_{c2}}\right),$$

where the emitter area ratios of Q_3_ to Q_1_ and Q_4_ to Q_2_ are both N = 24. Analyzing the collector current of each BJT, Q_1_ and Q_3_ are biased from the classic PTAT current (I_R1_ = ΔV_EB_/R_1_). However, the collector currents of Q_2_ and Q_4_ are mainly the temperature-insensitive current I_0_ mirrored by M_6_, which are changed by the compensation currents I_co1_ and I_co2_. Thus, the high-order temperature characteristics of ΔV_EB_ are changed.

The voltage V_fb_ is equal to V_ref_ because of the effects of the amplifier and NMOS source follower M_9_. The temperature-insensitive current I_0_ can be expressed as

(11) $$I_{0}\left(T\right)=\frac{V_{ref}}{R_{tb}\left(T\right)}=\frac{V_{ref}}{R_{tb}\left(T_{r}\right)\cdot \left[1+\alpha \left(T-T_{r}\right)\right]},$$

where the temperature also affects the resistance, the current obtained is not strictly temperature-independent. 

I_co1_ and I_co2_ are obtained by mirroring I_R1_, and are written as

(12) $$\left\{ \begin{array}{l} I_{co1}=y_{1}\cdot I_{R1}=\frac{{\left(W/L\right)}_{10}}{{\left(W/L\right)}_{9}}\cdot \frac{{\left(W/L\right)}_{7}}{{\left(W/L\right)}_{1}}\cdot I_{R1}+z_{trim1}\cdot I_{R1}\\ I_{co2}=y_{2}\cdot I_{R1}=\frac{{\left(W/L\right)}_{8}}{{\left(W/L\right)}_{1}}\cdot I_{R1}-z_{trim2}\cdot I_{R1} \end{array}\right.,$$

where y_1_ and y_2_ are the size ratios between the corresponding metal-oxide semiconductors. The parameters z_trim1_ and z_trim2_ are set by the trimming module in Figure 6, and the parameters y_1_ and y_2_ are adjusted to change the temperature drift of the output. Based on I_R1_ and I_0_, the corresponding I_c1_, I_c2_, I_c3_, I_c4_ and parameters are expressed as

(13) $$\left\{ \begin{array}{l} I_{c1}=I_{R1}\\ I_{c3}=b\cdot I_{R1}=\frac{{\left(W/L\right)}_{3}}{{\left(W/L\right)}_{1}}\cdot I_{R1}\\ I_{c2}=x_{1}\cdot I_{0}-I_{co1}=\frac{{\left(W/L\right)}_{2}}{{\left(W/L\right)}_{6}}\cdot I_{0}-y_{1}\cdot I_{R1}\\ I_{c4}=x_{2}\cdot I_{0}+I_{co2}=\frac{{\left(W/L\right)}_{4}}{{\left(W/L\right)}_{6}}\cdot I_{0}+y_{2}\cdot I_{R1} \end{array}\right..$$

The drain currents of M_3_, M_8_, M_10_ and M_11_ are obtained by mirroring M_1_, those of M_2_ and M_4_ are mirrored from M_6_, and the parameters b, x_1_, x_2_, y_1_ and y_2_ are the scale coefficients. By substituting (13) into (10), we obtain

(14) $$\Delta V_{EB}=V_{T}\left[2\ln\left(N\right)+\ln\left(b\right)\right]+V_{T}\ln\left(\frac{x_{2}\cdot I_{0}+y_{2}\cdot I_{R1}}{x_{1}\cdot I_{0}-y_{1}\cdot I_{R1}}\right).$$

Assuming that x_2_ = cx_1_ and y_2_ = cy_1_, where c is a constant. (14) can be rewritten as

(15) $$\begin{array}{ll} \Delta V_{EB} & =V_{T}\left[2\ln\left(N\right)+\ln\left(b\right)+\ln\left(c\right)\right]+V_{T}\ln\left(\frac{1+y_{1}/x_{1}\cdot I_{R1}/I_{0}}{1-y_{1}/x_{1}\cdot I_{R1}/I_{0}}\right)\\ & =\underset{\mathrm{linear}}{\underbrace{V_{T}\ln\left(bcN^{2}\right)}}+\underset{\mathrm{nonlinear}}{\underbrace{V_{T}\ln\left(\frac{1+h\cdot T}{1-h\cdot T}\right)}} \end{array}.$$

In Equation (15), it can be seen that the curvature compensation term shown as F_2_(T) in Equation (8) is introduced into ΔV_EB_ to compensate for the nonlinearity of V_EB_. Once the desired ΔV_EB_ is obtained, I_R1_ is mirrored by M_5_, and the resulting V_bgr_ is expressed as


(16) $$\begin{array}{ll} V_{\mathrm{bgr}} & =V_{EB}+a\cdot \frac{R_{2}}{R_{1}}\Delta V_{EB}\\ & =V_{EB}+a\cdot \frac{R_{2}\left(T\right)}{R_{1}\left(T\right)}\left[V_{T}\ln\left(bcN^{2}\right)+V_{T}\ln\left(\frac{1+y_{1}/x_{1}\cdot I_{R1}/I_{0}}{1-y_{1}/x_{1}\cdot I_{R1}/I_{0}}\right)\right] \end{array}.$$

Substitute (16) into (9) to obtain the final reference voltage V_ref_.

### 2.4. Process Variations and Trimming

The BJTs, resistances, and current mirrors in the proposed BGR circuit are the main sources of error owing to process variations and mismatches. BGR error sources are classified into two types: PTAT and non-PTAT errors. The errors caused by the spread of BJT saturation current and resistances R_1_ and R_2_ are mainly of the PTAT type. The BJT current gain spread, BJT base resistance, opamp offset, and BJT collector current mismatches mainly constitute the non-PTAT errors.

**Figure 7:**
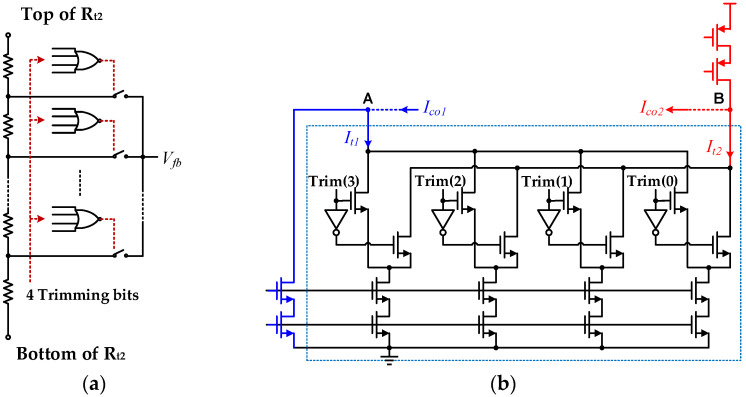
Schematic of the proposed trimming structure in BGR circuit: (**a**) resistance R_t2_ Trimming; (**b**) compensation current trimming.

PTAT errors are easily eliminated; thus, non-PTAT errors often determine the achievable precision of the BGR and require additional structures to ensure circuit accuracy. The proposed circuit contains many current mirror structures to provide bias and compensation currents for different BJTs. To minimize mismatches in the current mirrors, cascode-type current mirrors are used in the circuit to improve precision. In addition, the non-PTAT errors caused by process changes affect the proposed high-order curvature compensation method. The proposed trimming structure is shown in Figure 7.

Figure 7a is a 4-bit trimming resistance network [16] for I_0_ scaling, which is mainly used to ensure that the I_0_ change caused by the resistance spread does not affect the curvature compensation precision. At the same time, the change of I_0_ will also change the parameters c in (16). Figure 7b also depicts a 4-bit trimming structure for scaling the compensation currents I_co1_ and I_co2_ in the proposed circuit. This trimming structure is connected to the two nodes A and B shown in Figure 6 to change the parameters y_1_/x_1_ in (16) and achieve curvature compensation trimming. The two trimming blocks ensure appropriate curvature compensations in the presence of process variations or different application requirements. There are also trimming resistance R_t1_ connected to the output to adjust the reference voltage V_ref_. All trimming signals are generated by a fuse module controlled by the digital unit in our BMIC chip.

## 3. Experimental Results

**Figure 8:**
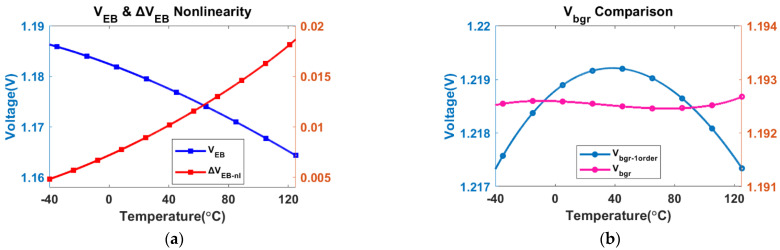
Simulated results of (**a**) V_EB_ and ΔV_EB_ versus temperature; (**b**) the first-order and proposed compensation of V_bgr_ versus temperature.

Firstly, the temperature characteristics of some key points are analyzed based on the simulation results. Figure 8a presents the simulation results of V_EB_ and ΔV_EB_ with temperature changes. The nonlinear ΔV_EB_ and V_EB_ present complementary slope trends in the range of −40 to 125 °C.

The bandgap voltage V_bgr_ before and after curvature compensation is shown in Figure 8b. V_bgr-1order_ is a first-order compensation result achieved after removing nonlinear compensation. The simulated results reveal that the maximum and minimum differences in V_bgr_ are reduced from 2 mV (without curvature compensation) to 0.2 mV (with curvature compensation) in the range of −40 to 125 °C. The best-found TC of V_bgr_ is 0.7 ppm/°C in the simulation result shown in Figure 8b.

**Figure 9:**
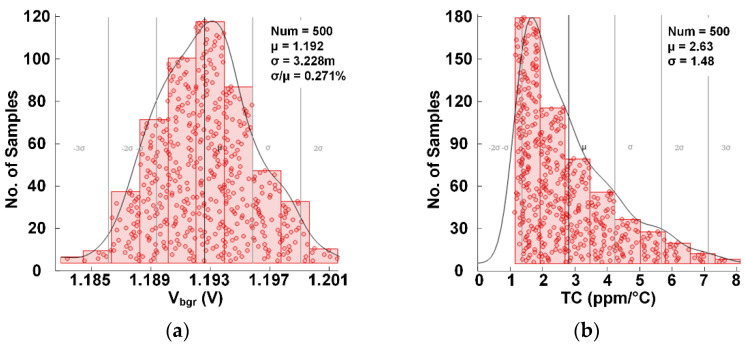
Statistics of untrimmed Vbgr from a 500-run Monte-Carlo simulation. (**a**) V_bgr_ @ 27 °C; (**b**) TC in ppm/°C of V_bgr_.

Figure 9 presents the 500 runs Monte Carlo (MC) simulation results of the proposed BGR with a 5 V supply voltage. The variation (σ/μ) of the reference voltage from MC results is 0.271% in Figure 9a. In Figure 9b, the statistical distribution of the TCs indicates that the average TC is 2.63 ppm/°C and the standard deviation is 1.48 ppm/°C. The MC simulation results show that the circuit is insensitive to mismatch.

**Figure 10:**
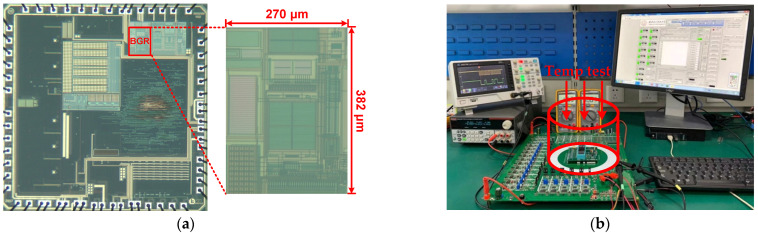
(**a**) Chip microphotograph of the proposed BGR circuits; (**b**) Photo of the BMIC test platform.

Figure 10a presents the chip microphotographs of proposed high-precision BGR circuit in the designed BMIC, which was implemented in a 0.18 μm BCD process. The whole BMIC area is 2.8 × 2.8 mm^2^, and the BGR circuit occupies a chip area of 0.38 × 0.27 mm^2^. We designed a BMIC test circuit to test the temperature and other related characteristics of the reference voltage of the chip. In the test circuit, place the BMIC in a white circle whose size corresponds to the cover of the temperature controller, isolated from other power supply and control modules, as shown in Figure 10b. This is to independently test the BMIC while simulating rapid temperature changes, ensuring accurate testing. At the same time, on the PCB, the relevant signals are connected to the outside of the white circle to ensure that the reference voltage changes can be monitored without affecting the temperature test.

**Figure 11:**
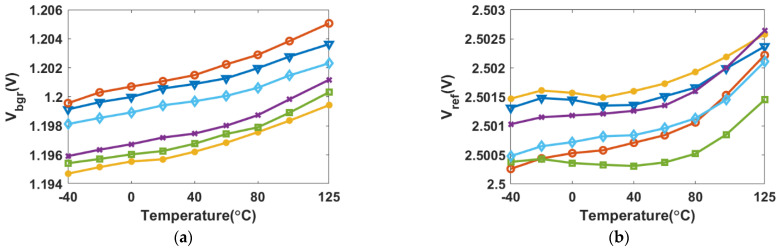
Measured TCs of six samples: (**a**) untrimmed V_bgr_ as a function of temperature; (**b**) trimmed V_ref_ as a function of temperature.

Figure 11 presents the measured temperature dependence of the bandgap voltage V_bgr_ and the reference voltage V_ref_ from −40 to 125 °C for six chips. The untrimmed V_bgr_ results are shown in Figure 11a. Process deviations cause the BGR output voltage to exhibit a positive temperature sensitivity, with an average TC of 26.04 ppm/°C. Figure 11b presents the measured V_ref_ results after TC trimming and voltage magnitude trimming. The tested optimal and worst TCs were 2.56 and 4.75 ppm/°C, respectively, and the σ/μ of the reference voltage is 0.11% at room temperature. The untrimmed inaccuracy is about ±0.43% (the maximum and minimum difference of V_bgr_ is 10.3 mV) over a temperature range of 165 °C, which decreases to approximately ±0.05% (the maximum and minimum difference of V_ref_ is 2.4 mV) after trimming at ambient temperature.

**Figure 12:**
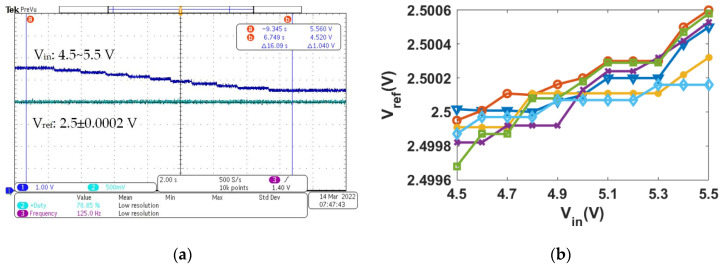
Measured reference voltage versus supply voltage: (**a**) oscilloscope monitoring results; (**b**) six chip measurement results.

In Figure 12a, it can be seen that the V_ref_ remains stable by continuously reducing the input voltage from 5.5 V to 4.5 V. Figure 12b presents the variation of six reference voltages versus supply at room temperature. When the supply voltage is increased from 4.5 to 5.5 V, the average variation in V_ref_ is 0.58 mV. Therefore, the average line sensitivity is 0.023%/V.

Table 1 summarizes the performances of the proposed BGR and compares it with some state-of-the-art designs. Compared to other BGR circuits, the proposed design achieves excellent temperature insensitivity, with a TC of 2.56 ppm/°C in the range of −40 to 125 °C. The current consumption of BGR is 53 µA with a 5 V supply voltage, and the area of the fabricated BGR circuit is 0.103 mm^2^.

## 4. Conclusions

A 2.5 V, 2.56 ppm/°C high-order curvature-corrected BGR over a temperature range of −40 to 125 °C is presented herein and implemented using 0.18 μm BCD technology. The circuit is based on the classic BGR structure using currents of different ratios and TCs to bias two sets of BJT pairs, thereby introducing nonlinear terms with compensation effects in ΔVEB to achieve temperature-independent voltage. The proposed structure is suitable for high-precision battery monitoring applications, such as EVs, energy storage, etc. Furthermore, this circuit has been used in the designed BMIC.

## Figures and Tables

**Table 1:** Performance summary and comparison with other works.

	[17] TCASII	[16] TCASII	[14] TCASI	[13] TCASI	[21] TCASI	[22] JSSC	This Work
Tech (μm)	0.18	0.18	0.18	0.13	0.18	0.16	0.18
Year	2021	2019	2017	2015	2014	2011	2022
Supply Voltage (V)	1.2–2.4	3.5–5	5.2	1.2	1.2	1.8	5
Reference Voltage (V)	0.628	3.11	3.65	0.735	0.767	1.088	2.5
Temperature Range (°C)	−40~120	−40~130	−40~110	−40~120	−40~120	−40~125	−40~125
TC Range (ppm/°C)	2.5~5	4.6~7.6	±3@3σ	9.3	4.9	5~12	2.56~4.75
LS (%/V)	0.03	0.031	N/A	N/A	0.54	0.48	0.023
Power (μA)	64.2	108	750	120	36	55	53
PSRR (dB)	−91.4 *	−92 *	−127	N/A	−80	−74	−84 *
Area (mm^2^)	0.024	0.223	0.28	0.063	0.036	0.12	0.103

* Simulation results.
